# Supplementary material for: Effects of exogenous melatonin on sugar and organic acid metabolism in early-ripening peach fruits
Source: PLoS One. 2023 Oct 13;18(10):e0292959. doi: 10.1371/journal.pone.0292959 (PMC10575493; doi:10.1371/journal.pone.0292959)
Supplement: Attachment — Submitted filename: Review.doc [file pone.0292959.s002.doc]

Reviewers' comments:

Reviewer's Responses to Questions

**Comments to the Author**

1. Is the manuscript technically sound, and do the data support the conclusions?

The manuscript must describe a technically sound piece of scientific research with data that supports the conclusions. Experiments must have been conducted rigorously, with appropriate controls, replication, and sample sizes. The conclusions must be drawn appropriately based on the data presented.

Reviewer #1: Yes

RESPONSE: Thank you for your reviewing.

2. Has the statistical analysis been performed appropriately and rigorously?

Reviewer #1: Yes

RESPONSE: Thank you for your reviewing.

3. Have the authors made all data underlying the findings in their manuscript fully available?

The [PLOS Data policy](http://www.plosone.org/static/policies.action#sharing) requires authors to make all data underlying the findings described in their manuscript fully available without restriction, with rare exception (please refer to the Data Availability Statement in the manuscript PDF file). The data should be provided as part of the manuscript or its supporting information, or deposited to a public repository. For example, in addition to summary statistics, the data points behind means, medians and variance measures should be available. If there are restrictions on publicly sharing data—e.g. participant privacy or use of data from a third party—those must be specified.

Reviewer #1: Yes

RESPONSE: Thank you for your reviewing.

4. Is the manuscript presented in an intelligible fashion and written in standard English?

PLOS ONE does not copyedit accepted manuscripts, so the language in submitted articles must be clear, correct, and unambiguous. Any typographical or grammatical errors should be corrected at revision, so please note any specific errors here.

Reviewer #1: Yes

RESPONSE: Thank you for your reviewing.

5. Review Comments to the Author

Please use the space provided to explain your answers to the questions above. You may also include additional comments for the author, including concerns about dual publication, research ethics, or publication ethics. (Please upload your review as an attachment if it exceeds 20,000 characters)

Reviewer #1: The research presented in the manuscript has investigated the effect of foliar supplied melatonin on metabolic changes in sugars and organic acids in peach fruit. Scientific claims made in the title, abstract and conclusion are justified with the graphical data presented in the manuscript. Organization, experimental design, and write up of the manuscript is fairly good. Just a little note to provide full name of the abbreviation when used first time in the manuscript. In short, manuscript do not need major revision and is good enough to be considered for publication in the journal.

RESPONSE: Thank you for your reviewing. We have revised for providing full name of the abbreviation when used first time.

6. PLOS authors have the option to publish the peer review history of their article ([what does this mean?](https://journals.plos.org/plosone/s/editorial-and-peer-review-process#loc-peer-review-history)). If published, this will include your full peer review and any attached files.


If you choose “no”, your identity will remain anonymous but your review may still be made public.


**Do you want your identity to be public for this peer review?** For information about this choice, including consent withdrawal, please see our [Privacy Policy](https://www.plos.org/privacy-policy).

Reviewer #1: No

RESPONSE: Thank you for your reviewing.
